# Keeping Active with Texting after Stroke (KATS): development of a text message intervention to promote physical activity and exercise after stroke

**DOI:** 10.1186/s40814-023-01326-x

**Published:** 2023-06-23

**Authors:** Linda Irvine, Jacqui H. Morris, Stephan U. Dombrowski, Jenna P. Breckenridge, Albert Farre, Gozde Ozakinci, Thérèse Lebedis, Claire Jones

**Affiliations:** 1 School of Health Sciences, University of Dundee, Dundee, UK; 2 Faculty of Kinesiology, University of New Brunswick, Fredericton, New Brunswick, Canada; 3 Faculty of Natural Sciences, Division of Psychology, University of Stirling, Stirling, UK; 4 NHS Grampian, Woodend Hospital, Aberdeen, UK; 5 School of Medicine, University of Dundee, Dundee, UK

**Keywords:** Co-design, Intervention development, Behaviour change, Text message intervention, Stroke, Rehabilitation

## Abstract

**Background:**

Post-stroke physical activity reduces disability and risk of further stroke. When stroke rehabilitation ends, some people feel abandoned by services and struggle to undertake physical activities that support recovery and health. The aim of this study was to codesign a novel text message intervention to promote physical activity among people with stroke and provide support when formal rehabilitation ends. This manuscript describes the intervention development processes that will inform future pilot and feasibility studies.

**Methods:**

The planned intervention was a series of text messages to be sent in a predetermined sequence to people with stroke at the end of rehabilitation. The intervention, underpinned by behaviour change theory and using salient behaviour change techniques, would provide daily messages offering encouragement and support for the uptake and maintenance of physical activity following stroke. The intervention was codesigned by a Collaborative Working Group, comprised of people with stroke, rehabilitation therapists, representatives from stroke charities and academics. A four-step framework was used to design the intervention: formative research on physical activity post-stroke, creation of the behaviour change text message intervention, pre-testing and refinement. Formative research included a review of the scientific evidence and interviews with community-dwelling people with stroke. Data generated were used by the Collaborative Working Group to identify topics to be addressed in the intervention. These were mapped to constructs of the Health Action Process Approach, and salient behaviour change techniques to deliver the intervention were identified. The intervention was rendered into a series of text messages to be delivered over 12 weeks. The draft intervention was revised and refined through an iterative process including review by people with stroke, their spouses, rehabilitation therapists and experts in the field of stroke.

The messages encourage regular physical activity but do not prescribe exercise or provide reminders to exercise at specific times. They use conversational language to encourage engagement, and some are personalised for participants. Quotes from people with stroke provide encouragement and support and model key behaviour change techniques such as goal setting and coping planning.

**Discussion:**

Co-design processes were critical in systematically developing this theory and evidence-based intervention. People with stroke and rehabilitation therapists provided insights into perceived barriers post-rehabilitation and identified strategies to overcome them. The structured multistep approach highlighted areas for improvement through successive rounds of review. The intervention will be tested for acceptability, feasibility and effectiveness in future studies. This co-design approach could be used for interventions for other heath behaviours and with different populations.

**Supplementary Information:**

The online version contains supplementary material available at 10.1186/s40814-023-01326-x.

## Key messages regarding feasibility

What uncertainties existed regarding the feasibility?

- Would people with stroke engage with a behaviour change intervention delivered by text message?
- What content and form of delivery would be most useful for increasing physical activity?
- Would it be feasible to identify and recruit people at the end of stroke rehabilitation?

What are the key feasibility findings?

- Co-design with people with stroke, therapists and experts in stroke rehabilitation were essential for developing this theoretically and empirically informed text message intervention
- People with stroke confirmed they would welcome an intervention at the end of rehabilitation.
- Successive rounds of review and revision by the co-design team led to a coherent intervention to be delivered over 12 weeks.

What are the implications of the feasibility findings for the design of the main study?

The feasibility and acceptability of the intervention will now be tested in a pilot randomised controlled trial. Areas for consideration are as follows

- Working with therapists to recruit people from different community settings
- Using accelerometry as the primary outcome measure of physical activity and determining appropriate secondary outcomes
- Conducting interviews for qualitative evaluation of acceptability and exploring how participants used the intervention to inform further refinement

## Background

Regular physical activity following stroke contributes to substantial health benefits including improved fitness, balance and walking ability [1–5] and may reduce factors such as hypertension and raised cholesterol levels that increase the risk of recurrent stroke [6, 7]. Despite this, many people with stroke are classified as sedentary [8–11], placing them at increased risk of health problems [12].

A range of physical activities and exercises to improve strength, balance, motor control and function and aerobic fitness are essential for recovery from stroke [4, 13, 14]. Internationally, early guided rehabilitation exercise programmes focus on intensive, repetitive task-orientated training supervised by a physiotherapist [13–15]. When rehabilitation ends, people with stroke often feel abandoned by support services [16–19] and marginalised due to a lack of continuity of care and access to community services [20]. To ensure long-term benefit, people with stroke need to move from structured exercise plans to self-directed activities within their own environment [21]. Low levels of physical activity in the year after stroke are high among people living in the community [9–11], where recorded daily step counts may be half of those of age-matched healthy people [8]. Longitudinal data show that low post-rehabilitation physical activity levels remain so over the subsequent 2 years [8, 22]. Beyond formal rehabilitation, many face challenges in achieving recommended levels of physical activity, including modifiable factors such as physical function, low self-efficacy, fatigue, fear of falls and depression [23]. People may feel they do not have the knowledge and resources to continue with exercise and activities to enhance their recovery [20, 24].

One resource-efficient way to promote physical activity is via text messaging. Text message interventions can provide structured support, embedding evidence-based behaviour change theory and techniques into a series of messages, to guide people through the process of change. Text messaging is suited to people with stroke, some of whom may have cognitive problems or upper limb weakness which may affect the ability to use complex digital interventions. Text messaging requires little effort to engage, and participants do not have to learn how receive or use the intervention [25, 26]. Interventions which require participants to login or access a website or application present barriers for many [27].

Interventions using mobile phones have been used widely to promote physical activity in general populations with meta-analyses reporting modest effects on objectively measured activity levels [28–32]. A recent meta-analysis reported a statistically significant higher post-intervention daily step count (Cohen’s *d* = 0.38, 95% *CI* = 0.19, 0.58, based on 10 studies) [31], although the effect on moderate to vigorous physical activity was not statistically significant. A few pilot studies have reported the potential for text message interventions to promote physical activity among people with stroke [33–36]. The STROKEWALK study delivered instructional text messages to promote regular walking and functional leg exercises over 3 months [36]. A study by Kamwesiga et al. reported early pilot work, where reminder text messages to perform three daily activities were used as part of a family-centred intervention to support participation in daily activities [34, 35]. These mainly provide instruction or prompts to undertake activity, rather than sequenced behaviour change strategies and have yet to be tested in randomised controlled trials. Cadhilac et al. have also developed the iVERVE intervention which uses text messages, informed by behavioural *theory and behaviour change techniques*, as part of a self-management programme to support goal attainment for recovery after stroke and in secondary prevention after stroke [33]. These studies vary in terms of the level of involvement of people with stroke and other stakeholders during development. An iterative and person-centred approach is recommended and is essential for developing and evaluating digital interventions [37].

The objectives of this study were as follows: (1) to codesign with people with stroke, their family/friends and rehabilitation professionals, a novel, theoretically informed behaviour change intervention, comprising a series of text messages, to support community-dwelling stroke survivors to adhere to physical activity and goals for recovery, and (2) to determine what community-dwelling stroke survivors consider to be key features of a texting intervention to ensure that it is accessible, acceptable and useful in supporting their post-rehabilitation exercise and PA goals.

This manuscript therefore describes the intervention development processes that will inform future pilot and feasibility studies. It presents the systematic development of a co-designed novel, theoretically informed text message intervention to support community-dwelling people with stroke to be physically active when formal rehabilitation ends. The process involved multistage, iterative development with contributions from people with stroke, health professionals and experts in the field of stroke rehabilitation. The messages are underpinned by behaviour change theory, the Health Action Process Approach (HAPA) [38], and use established behaviour change techniques [39] to provide support and guidance for increasing and maintaining regular activity. Early messages, to foster interest and engagement, are followed by a series of messages to address and illustrate the process of behaviour change in sequence: increase motivation and create intentions to change, encourage commitment to setting goals for physical activity going forward, make action plans, identify barriers to being active and develop coping strategies and increase self-efficacy for long-term maintenance.

## Methods and results

Ethical approval was granted by the University of Dundee Schools of Nursing & Health Sciences and Dentistry Research Ethics Committee (UOD/SHS/2020/022/MORRIS). Informed consent to participation was given by participants.

The intervention was developed in line with the UK Medical Research Council Framework for Complex Interventions [40], using iterative co-design processes and informed by a framework for developing text messaging programmes created by Abroms et al. [41]. The four-step framework covered formative research on physical activity among people with stroke, the design of a theoretically and empirically informed text message intervention, pre-testing of the intervention and subsequent revision of the text messages (Table 1).

**Table 1:** Framework for intervention design

*Component of intervention design co-design partners*	*Co-design partners*
**Step 1: Formative research**	
1.1. Survey to assess mobile phone use following stroke	People with stroke
1.2. Review of the scientific evidence a. Barriers and facilitators for physical activity after stroke b. National and international guidelines for rehabilitation and physical activity following stroke c. Tailoring text message interventions for the target group	Core Research Team (CRT)
1.3. Clarifying what people with stroke want/need from an intervention a. Addressing perceived gaps in services b. Ensuring continuity from rehabilitation c. Building on rehabilitation goals d. Exploring what input would be helpful in an intervention	Collaborative Working Group (CWG)
1.4. Interviews with community-dwelling people with stroke to assess: a. Acceptability of a text message intervention post-rehabilitation b. Useful content for the intervention c. Design features to tailor the intervention to the target group	Community-dwelling people with stroke
1. 5. Identify theoretical models for behaviour change	CRT
**Step 2: Design of the text message intervention**	
2.1. Defining intervention content a. Map out components of the intervention, using theoretical models b. Identify salient behaviour change techniques (BCTs) c. Tailor the intervention to the needs of people with stroke d. Create an initial bank of messages based on step 1 data	CRT
2.2. Refining the content and form of delivery for people with stroke a. Giving a voice to people with stroke within the messages b. Incorporating recommended guidelines for post-stroke activity c. Recommendations on use of online resources	CWG PPI group
2.3. Creating draft 1 of the text message intervention a. Organising messages into a sequence for delivery following the Health Action Process Approach (HAPA) and the maintenance model b. Creating a delivery plan for the intervention c. Ensuring messages are coherent and deliver the intervention	CRT
**Step 3: Pretest the intervention concept and messages**	
3.1. Review draft 1 to ensure components of the intervention are addressed and behaviour change techniques are incorporated	CRT
3.2. Revision of messages and delivery plan (draft 2)	CRT
3.3. Review of draft 2 to ensure readability for people with cognitive and visual processing difficulties	Speech and language expert
3.4. Review of draft 2 to assess acceptability of content and delivery schedule, identify gaps in the intervention, ensure that messages are engaging, understandable, interesting and informative	CWG, people with Stroke, other volunteers
**Step 4: Final revisions**	
4.1. Review and final revisions to the intervention a. Collate all feedback from step 3 b. Create final version of the text message intervention and delivery plan for pilot testing	CRT

Prior to obtaining research funding, a meeting was convened with the research team’s patient and public involvement (PPI) group, which included five people with stroke and three spouses. The group supported the development of a text message intervention for people who were at the end of rehabilitation following stroke. They identified challenges that people with stroke experience in maintaining regular physical activity at this time of transition. The group felt that a text message intervention would be a simple but important way to offer encouragement, increase motivation and pass on tips from other people with stroke.

The Core Research Team (CRT) codesigned the intervention with a Collaborative Working Group (CWG) [42]. The GWC uses a structured approach for bringing together key individuals to support collaborative decision-making and iterative intervention refinements. To create the CWG, a list of the expertise required to develop the intervention was drawn up. People with stroke who had already advised on the team’s research projects were invited by email and telephone call to take part (face-to-face contact was not permitted due to COVID-19 restrictions). They were also given written information about the format of the meetings and what their input might be. Rehabilitation therapists from two health boards were invited by email to take part. Their knowledge and insight into potential participants’ needs would be essential in identifying potential content for the intervention and for planning a recruitment strategy. Academic colleagues with expertise in stroke rehabilitation, intervention design and trial management were then recruited by email invitation. Finally, stroke charities were approached by email to invite representatives involved with people undergoing post-stroke rehabilitation.

The CWG included eight people with stroke from existing PPI groups, seven rehabilitation therapists, eight academics (with special interest in stroke rehabilitation, biomechanics, clinical trials and speech and language therapy) and two representatives from stroke organisations, i.e. Chest Heart and Stroke Scotland and the Stroke Association. The CWG met on two occasions, through videoconferencing, rather than the intended face-to-face meetings, due to restrictions imposed by the COVID-19 pandemic. The size of the group aimed to ensure as wide a range of stakeholders as possible whilst ensuring the group was not too large for meaningful discussion within the videoconferencing format. An additional online meeting was convened with the PPI group, and a subject expert in goal setting in rehabilitation was consulted on one occasion. One member of the PPI group was part of the CRT. See Table 1 for details of how the co-design methodology was incorporated into Abroms et al.’s four-step framework [41].

### Co-design of the text message intervention

#### Step 1: Formative research

***Step 1.1: Survey to assess mobile phone use following stroke*** Physiotherapists from one Scottish Health Board were invited to conduct a short, anonymised survey with community-dwelling clients who were receiving post-stroke rehabilitation to assess the feasibility of a text messaging intervention. The sample was a convenience sample, based on the cohort of physiotherapists in the area and on the patients in their care who were able to respond. Forty-nine people provided information about their mobile phone ownership and use.

Most respondents (*n* = 42, 86%) owned a mobile phone, with another 8 having access to a spouse’s phone. Of those who owned a mobile phone, 31 (63%) owned smartphones. Whilst 38 people (77%) received text messages on their phone, 35 (71%) used their phone to send messages. Around half of respondents (*n* = 25, 51%) used a phone to access the Internet or applications.

***Step 1.2: Review of the scientific evidence*** To ensure that the intervention was informed by relevant and contemporary empirical evidence, three information sources were reviewed: (a) the scientific literature on barriers and facilitators to physical activity after stroke, (b) national and international guidelines for physical activity post-stroke and (c) tailoring text message interventions for the target group.

- Barriers and facilitators for physical activity after stroke

Systematic reviews and syntheses of barriers and facilitators to physical activity post-stroke identified topics to be addressed within the intervention [23, 43–49]. Common barriers to physical activity post-stroke include negative emotional responses to physical activity, such as embarrassment, fear of falling and fear of having another stroke, and physiological factors including fatigue, *impaired* physical function and low cardiorespiratory fitness [13, 23, 47, 50]. Perceptions of limited capability due to stroke, cognitive and communication impairments, low self-efficacy, mood, confidence and expectations of recovery contribute to inactivity after stroke. Facilitators for physical activity following stroke include self-determination to regain function and return to previous activity, enjoyment of being active, previous success in physical activity and social support [43, 47, 50–52].

b. National and international guidelines for rehabilitation and physical activity after stroke

Current international guidance for physical activity after stroke [13, 14, 21] suggests that people should be active every day and avoid long periods of sitting. They should aim to accumulate 150 min or more of moderate intensity physical activity per week and engage in muscle strengthening activities at least twice per week. The intensity of physical activity should be gradually increased. The guidelines provided suggestions on how physical activity could be increased, e.g. through setting meaningful goals for activities of daily living and leisure interests and participation in group social activities.

c. Tailoring text message interventions for the target group

Many studies and systematic reviews have identified features that may increase engagement and effectiveness of text message interventions [27, 28, 30, 31, 41, 49, 53–56]. These include the use of psychological theories, BCTs and tailoring of interventions for the target group or individuals. Other characteristics include the duration of interventions, personalisation of messages, interactive messages, the frequency of delivery of messages and the language used. It remains unclear which of these, or combinations of these, are most effective in achieving and sustaining behaviour change [26, 28, 31, 49, 56]. Understanding the target group’s circumstances and values can facilitate tailoring of content and delivery of an intervention, which increases receptiveness and leads to greater engagement [27, 49].

***Step 1.3. Clarifying what people with stroke want/need from an intervention*** To define the parameters of the intervention and inform its development, the CWG met to assist in interpreting data from Steps 1.1 and 1.2. A CWG uses a structured reflective process of “what, so what, now what?” to guide collaborators in collectively analysing emerging findings, making decisions about intervention design and informing the direction of the ongoing research [42]. The CWG considers the following: (a) *What* do the data tell us? (b) *So what* does this mean for the intervention? and (c) *Now what* information do we need to refine the intervention,and how will we adapt our research design? CWG meetings were video-recorded and transcribed. Decisions taken by the group were collated and action points taken forward by the CRT. At the first CWG meeting, with 21 attendees and lasting 1 h 55 min, the group confirmed that people with stroke and therapists believe that the proposed intervention could complement community rehabilitation. Hospital and community therapists negotiated how they could support the delivery of a text message intervention by identifying potential participants at key stages in their recovery (on discharge from hospital or at the end of community rehabilitation). Together, therapists and people with stroke clarified how text messages could bring continuity from supported goal setting to participants setting goals independently. Therapists agreed that they could liaise with the study facilitator in advance of participants receiving the intervention, so that personalised information about recent goal setting and plans could be incorporated into messages.

***Step 1.4. Interviews with community-dwelling people with stroke*** Having established the relevance and potential usefulness of the proposed intervention, telephone interviews were conducted with 14 community-dwelling people in rural and urban areas from two Scottish Health Boards. We adopted a purposeful sampling strategy to obtain a maximum variation sample, based on sex, time post-stroke and sociodemographics. We based the sample size on our previous work in intervention development (46) that indicated that a sample of 12–14 participants would provide adequate information to inform intervention development. We also analysed the transcripts as the study progressed and completed sampling when we saw that no new ideas were emerging. The aim of the interviews was to ascertain participants’ opinions on the acceptability and feasibility of a text message intervention and to explore topics to be addressed in the messages. Prior to the interviews, participants were given 15 illustrative text messages, presented in a word document. Text messages were displayed on images of a mobile phone, to demonstrate the possible format and content of the proposed intervention.

*Recruitment*

Potential participants were identified by therapists and from local stroke groups and stroke exercise classes. Nine men and five women, aged from 37 to 79 years (median 57.5 years), were recruited. The time since stroke ranged from 4 months to 16 years. Participants provided representation across the sociodemographic spectrum. The Scottish Index of Multiple Deprivation (SIMD) [57] scores for participants included all quintiles: numbers per quintile, 1 to 5 (1 being the most disadvantaged), were 6, 1, 2, 2 and 3, respectively.

*Data collection and analysis*

Participants were asked to reflect on their rehabilitation period, including the types of physical activity goals that were set during rehabilitation and beyond and personal barriers and facilitators for being active. They discussed their readiness at the end of the rehabilitation period for an intervention to increase physical activity. They gave comments and suggestions on the illustrative text messages including intervention components, content of the messages, language used, timing and frequency of message delivery and duration of the intervention.

Telephone interviews, conducted by the study researcher (LI), were recorded and transcribed. Field notes were taken to provide context. The six stages of thematic analysis [58] were used by the research team to interpret findings and inform intervention development. The transcripts were read by two researchers (L. I. and J. M.) to identify a coding framework for organising the data. The initial codes were reviewed, agreed and grouped into categories relevant to the description and evaluation of participant perceptions of intervention components. Interpretation of the data was agreed with the rest of the research team. Data were managed in NVivo V.12.

*Findings*

*Usefulness of messages* Almost all participants (*n* = 12/14) reported that they would have welcomed the opportunity to receive a text message intervention at the end of their rehabilitation. Most had been able to use their phones early in their recovery and felt that most people would have the ability to engage in the intervention by the time their rehabilitation was complete. Some felt that an intervention would have provided continuity of support at a time when they felt vulnerable. The interviews confirmed that some people with stroke feel abandoned by services at the end of rehabilitation and experience isolation:

All of a sudden, I seemed to have just got dangled over a cliff and dropped (02, male)

I felt it was a big world out there and I needed all the help I could get (03, female)

Participants believed that text messages may be able to reduce feelings of abandonment and isolation by keeping in touch:

To get people reminded that they’re not lost in the system, that although things have changed that they’re no longer being seen, they’re not lost in the system (09, male)

Just getting that text would be enough, no matter what it said (05, female)

Participants were aware that the end of active rehabilitation can lead to a dip in motivation to be physically active and saw the proposed intervention as a way to boost motivation at that crucial time:

The important thing is to keep people motivated and I don’t know whether they’ll be motivated. People will motivate themselves or the stroke will be so devastating they won’t feel like motivating themselves (03, female)

Anything that would give me tips and tricks on what to do to improve, I think would be helpful (13, male)

Some participants talked about cognitive problems, visual processing difficulties and a need to avoid information overload. Succinct easy to read and process messages were preferred.

It’s easy to read the texts…… My brain gets fed up trying to read it, if it’s too much information (11, male)

*Terminology and tone* The sample messages were informal, in keeping with texting etiquette, and a non-authoritative style was used throughout. Participants welcomed the friendly tone as it fitted with the medium being used:

I think for a text that is appropriate because texting is quite informal (03, female)

I would prefer if there was more off the cuff and off-hand, rather than being terribly formal (04, male)

The messages provided encouragement and avoided prescribing physical activity. Participants felt strongly that the messages should encourage but not dictate when to exercise:

I don’t like getting told what to do as I said, but getting encouraged is okay, there’s nothing wrong getting encouraged (02, male)

And if you get a text to say, “Oh, you should really go for a walk today” and it’s pouring with rain—you would just throw the phone away (05, female)

Participants clarified terms for physical activity and exercise that would be most appropriate:

Not everybody sees exercise as being in the gym, some would maybe get put off by that, so, the general thought on that would have been not to refer to it as exercise but refer to it as activity (10, male)

***Step 1.5. Theoretical models of behaviour change to underpin the intervention*** To consolidate the insights from the extant literature, CWG expertise and interviews, the Health Action Process Approach (HAPA) [38] was selected to guide intervention development. The HAPA provides a theoretical framework outlining the processes required to change behaviour. It involves two phases: a motivational phase where the intentions to change are formed (influenced by perceptions of risk, outcome expectancies and action self-efficacy) and a volitional phase where intentions are translated into behaviour through action planning, coping planning and self-efficacy. The HAPA provides a framework for integrating effective behaviour change techniques (BCTs). BCTS are discrete components, e.g. goal setting, action planning and self-monitoring, used in combination at appropriate stages of the intervention delivery to facilitate behaviour change [59]. Selected relevant BCTs from Michie et al.’s taxonomy of 93 techniques were incorporated to operationalise the intervention [39]. The behaviour change maintenance framework [60] was used to ensure BCTs for sustained physical activity were included.

#### Step 2 Design of the text message intervention

***Step 2.1. Defining intervention content*** Data from the evidence reviews, interviews and team discussions were collated to identify topics to be addressed in the intervention. Table 2 lists topics for inclusion and the sources of information that informed their selection. An additional file describes in detail the challenges identified and solutions obtained through collaborative working (see Additional file 1). Topics for inclusion were systematically mapped to each of the HAPA constructs and themes for maintenance [60]. Next, salient BCTs to deliver the intervention, identified from the Michie et al. taxonomy [39], were mapped on to each HAPA construct (see Additional file 2). A logic model was produced to guide the development of the intervention and to clarify how behaviour change might be achieved. The logic model was used to guide the development of the intervention and to clarify how behaviour change might be achieved. The model is provided in Additional file 3.

**Table 2:** Topics to be addressed in the intervention

Topic	Sources of information^a^
Reduce feelings of abandonment and isolation	PPI Literature
Provide continuity from rehabilitation	CRT CWG
Encourage participants to continue with exercises and activities recommended by therapists and stroke nurses	CRT CWG
Encourage participants to work towards goals agreed with therapists at the end of rehabilitation	CRT CWG
Build on the goals agreed by therapists at the end of rehabilitation	CRT CWG
Move on to setting personal goals (without therapist input)	PPI CWG
Suggest setting goals for activities participants would enjoy (to increase maintenance)	PPI CRT HAPA
Encourage planning how to return to pre-stroke activities	CRT CWG HAPA
Provide examples of goals and plans	HAPA PPI
Use quotes from people with stroke: • To demonstrate they are not going through it alone • To model intervention components, e.g. goal setting	HAPA PPI Interviews
Encourage enlisting the support of family members and friends	HAPA Literature
Give examples of how family and friends can help	CRT Literature/guidelines
Address low self-efficacy	Literature PPI Interviews
Encourage people to walk (safely) outside	Literature/guidelines
Suggest engagement with online resources for physical activity	PPI CWG
Suggest self-monitoring of activities (provide a blank calendar)	PPI HAPA Literature
Encourage regular reflection on progress	HAPA Literature
Explain and model coping planning, e.g. what to do in bad weather, when fatigued, when exercising becomes tedious	HAPA PPI
Provide prompts to be active	Maintenance model PPI
Encourage habit formation	HAPA Maintenance model
Promote maintenance in the long term	Maintenance model CWG
Use humour to engage and maintain interest	Literature

^a^Abbreviations used in Table 2. *PPI* public and patient involvement; *CRT*, Core Research Team; *CWG*, Collaborative Working Group; *HAPA*, Health Action Process Approach

##### Writing the text messages

One member of the CRT (L. I.), with expertise in developing in text messaging interventions, wrote messages for each of the topics identified. Initially, a bank of more than 150 messages was constructed. These were tailored to address challenges people with stroke encounter and guide them through the behaviour change process. Message length was restricted to 160 characters which is the allowance for one text message. Texts were written in complete sentences. Texting abbreviations, which may be misinterpreted, were avoided as studies have shown that texting abbreviations may make intervention studies less credible [41]. Emojis were used sparingly, on days when humour was used.

Strategies to enhance acceptability of the messages and increase engagement were incorporated into the intervention [27, 41, 49] (Table 3). The interviewees (Step 1.4) suggested that it would be helpful if the messages appeared to come from a named individual. Credibility of the source increases engagement and receptiveness to an intervention [49]. To increase credibility, some messages each week were signed off by the study researcher. Messages containing quotes from people with stroke were used several times each week to provide encouragement and support, give hints and tips and to model key behaviours and BCTs such as goal setting or coping planning. Asking questions was used as a tool to promote cognitive engagement, interaction and increase retention. Questions were matched to HAPA constructs [38] and were framed in different ways to encourage engagement and enactment, e.g. to report physical activities undertaken, describe a goal or reflect on progress.

**Table 3:** Strategies used to enhance the acceptability of the intervention

Strategy	Purpose
Personalise messages with participant’s name a few times each week	Increase engagement
Add researcher’s name a few times each week (this would be a therapist for a large trial and/or at rollout of the intervention)	Increase engagement Provide credibility of the intervention Messages from humans are preferred
Mention the participant’s therapist name on two occasions during first 2 weeks	Provides continuity from rehab services Provides credibility of the intervention
Mention goals set with the therapist on discharge from rehabilitation	Provides continuity from rehab services Encourage participants to continue with and build on work done with the therapists
Use weekly themes, e.g. goal setting, self-monitoring, benefits of walking, coping planning and problem-solving, developing routines and habits	Provide structure and opportunity to develop a theme over the period, i.e. explain the concept, suggest how to do it, model the behaviour by examples from a person with stroke
Ask direct questions and rhetorical questions. Participants would be able to reply to messages (which would be received by the research team). However, they would not receive a reply in return	Increase engagement and interactivity Encourage reflection on progress
Use quotes from other stroke survivors (taken directly from interviews with people with stroke)	Provides credibility of the intervention Helps participants feel they are not the only one facing the challenges Encourage participants to try what others have done or consider what they could achieve
Use the voice of other people with stroke to pass on hints and tips about being active	Avoids didactic instruction. Interviewees made it clear that people did not want to be told what to do
Incorporate humorous trivia, usually loosely related to physical activity, to increase appeal to a heterogeneous group	Increase engagement, encourage interactivity Used at the weekend to help provide structure
Schedule message delivery between 10 am and 5:00 pm on weekdays and 10:45 am and 7:10 pm at the weekends	Avoid early mornings and evening as fatigue is often a problem. Some people have carers at those times
Incorporate guidelines for physical activity after stroke explained using a physiotherapists voice	Raise awareness of guidelines without telling people what they should be doing Provides credibility
Suggest online opportunities to be active suggested (credible sites recommended by therapists)	Provide credible options for different forms of physical activity

The daily routines and availability of the target group to interact with the intervention can affect acceptability and engagement [49]. Fatigue, a common post-stroke problem, was highlighted by the PPI group and interviewees (Step 1.4). Although text messages can be opened at the recipients’ convenience, most messages were scheduled to be sent between 10:30 am and 5:00 pm, when participants were likely to be most receptive and available to engage in physical activity. Within this window, messages were scheduled to be delivered at different times of the day to maintain an element of anticipation. Saturdays’ messages often included some trivia or humour, a technique used in previous studies, to provide some light relief after the more serious topics discussed during the week [61–63]. This emphasised the break between the weeks and encouraged relaxation and reflection at the weekends. People with stroke (Step 1.4 interviews) felt that humour was appropriate and would not detract from the key messages being delivered:

Aye well, a touch of humour never goes amiss, does it? (04, male)

***Step 2.2. Refining content and form of delivery for people with stroke*** Having created a bank of messages, a second CWG meeting to start the refinement process was convened. At an online meeting with 17 people, lasting 1 h and 50 min, the CWG explored whether additional materials should accompany the text messages, namely a handbook, a diary and a list of online resources. Some studies report that supplementary materials increase effectiveness [28]. As some people with stroke have trouble with memory and cognition, the CWG agreed that a study handbook would be helpful for operationalising the selected BCTs. It would provide additional information, reinforce topics addressed and explain key components of the intervention in greater detail. The CWG decided that a blank calendar for the intervention period should be provided. Participants would be invited to use it for setting goals, recording activities undertaken and monitoring and reflecting on progress. Current guidelines for physical activity after stroke recommend 150 min of moderate to vigorous activity per week. People with stroke gave mixed feedback on whether this information should be included. Some felt that it was so far removed from some people’s capability, it might be disheartening and therefore counterproductive. Others felt it gave hope and encouragement to strive to improve. To discuss in more detail, a separate meeting was convened with the PPI group. A decision to include it in the intervention was subsequently taken, but with guidance on how it could be done in manageable sessions, to made it appear less daunting. Data from the CWG and PPI meetings were collated for incorporation into the text messages.

***Step 2.3 Creating draft 1 of the text message intervention*** From the initial bank of messages, individual messages were mapped on to a 12-week blank calendar, to provide a coherent sequence. The intervention was a set of unique messages to be delivered to all participants as a discrete intervention. Although messages were stand-alone and did not require the reader to know what had gone before, the daily messages were designed to build on previous topics to guide the reader to sustained regular physical activity.

The intervention was organised in weekly blocks, following the HAPA structure, to guide participants from forming intentions to be active, to action through goal setting, planning, self-monitoring and coping planning and to achieve sustained physical activity. Participants were encouraged to set new goals at the beginning of the week, monitor their activities mid-week and then reflect on progress towards the end of the week. This reflection would feed into decisions about setting new goals for the next week.

Draft 1 comprised 134 messages to be delivered over 12 weeks. Messages were scheduled to be delivered on 5 or 6 days on most weeks. The complexity of the intervention meant that on some days, a few messages were required to deliver all the information. The first message would introduce the topic, e.g. goal setting, a second would demonstrate how to do it and a third would model the behaviour by giving an example of a goal used by another person with stroke.

#### Step 3 Pre-testing the intervention concept and messages

To prepare for pre-testing draft 1 of the intervention, the text messages and delivery plan were presented as a table in a word document, with columns containing the following: day of the week, time for message delivery, the message and a blank space for comments. The individual messages and delivery plan were reviewed iteratively. Draft 1 was initially reviewed by the CRT, and revisions were made (Step 3.1). Following revisions, draft 2 was reviewed by a speech and language expert (Step 3.2), people with stroke and their spouses, health professionals and experts in the field of stroke (Step 3.3). Reviewers gave feedback in one of three ways: by providing a summary of overall impressions and suggestions for change, by commenting on individual text messages or by detailing specific points that should be changed. Reviewers were asked to consider whether participants would engage with the intervention, whether they felt that the content would be helpful and if any important content was missing. Finally, they were asked to reflect on the tone of the messages, to identify messages that were unclear or interrupted the flow and to identify topics or specific information that should be included.

***Step 3.1. Review and revisions by the Core Research Team*** The remit for the CRT was to ensure that behaviour change components were adequately addressed at appropriate times using BCTs in line with the underpinning theory. Ensuring that participants will be engaged from the outset and that interest will be maintained for the duration of the intervention is paramount. A key decision taken at this stage was to reduce the number of messages in the intervention. A few people with stroke had indicated that they would not want too many messages. The number of messages was reduced to one per day, every day for 12 weeks, apart from 11 days when two messages were needed to develop a theme or model a particular behaviour. This reduction was achieved by increasing the number of characters in individual messages. Most people have smart phones, so this was seen to be acceptable. Although the maximum number of characters per message is 160 characters, smart phones facilitate concatenation, so that messages with more than 160 characters appear as one message, making them easy to read. For draft 2, messages had up to 390 characters which were broken down into short paragraphs for ease of reading. Thus, the intervention was reduced to 95 messages without losing content. Additional changes included more personalisation and adding messages in the voice of people with stroke and therapists to enhance credibility (Table 4).

**Table 4:** Strategies to enhance engagement

Restrict the number of messages sent in a day, to avoid overloading participants
Send a message every day, so participants know what to expect
Add a few more messages to establish a relationship with the participants over the first few days
Refer to the participants’ names more frequently within texts
Sign off more messages every week using the researcher’s name (this would be a therapist for a large trial and/or at rollout of the intervention)
Add more information in text messages that would provide continuity from participants’ personal rehabilitation packages
Add more texts with hints and tips as verbatim quotes from other people with stroke, to model specific behaviours to increase physical activity
Make the therapists’ voices stronger (including quotes from physiotherapists how to achieve recommended physical activity levels)
Place more emphasis on walking, as it is one of the best forms of physical activity post-stroke, and it can be interesting, easily accessible and free

***Step 3.2. Review and revision by speech and language expert*** Draft 2 was reviewed by a speech and language therapist who amended messages to ensure that the grammar was as simple as possible. Long sentences were shortened by removing words that were redundant and by simplifying phrases when it did not interrupt the flow or meaning of the messages. Apostrophe abbreviations were removed to maintain clarity for people with visual processing difficulties, although care was taken to maintain informality of the messages. Some sentences were expanded to ensure clarity and any complex phrasing simplified.

***Step 3.3. Review and revision by service users and experts in the field of stroke*** The updated messages (draft 2) were sent by post or email to 15 people for review. The reviewers comprised people with stroke from the CWG (*n* = 4), participants who were interviewed at Step 1.4 (*n* = 2), people with stroke who were participants in the team’s previous research (*n* = 2), spouses of participants from previous research (*n* = 3), physiotherapists (*n* = 3) and a Stroke Association Clinical Lecturer.

Suggestions from the review group included ensuring gender equality within the text messages and checking that the messages were appropriate for a heterogeneous group. Therapists felt that there was too much emphasis on walking and not enough suggestions for other types of activities, especially for people who are unable to walk outside. Other activities were subsequently added, e.g. home-based exercise, household chores and online classes. People with stroke and therapists requested more examples of goals for physical activity. This prompted an extra meeting with the PPI group to identify real-life goals and ways to achieve them, e.g. using clothes pegs to strengthen fingers and taking laundry out of the washing machine with the weak hand. All reviewers welcomed the quotes from other people with stroke. However, they pointed out that these should be not just the opinion of one person but relevant and helpful to the majority. Finally, people with stroke requested the addition of more practical tips. These included adding more information about coping with fatigue, how to incorporate exercises into everyday activities, reminders to use a stick or walking aid if required and wearing good shoes when doing activities.

#### Step 4 Final revisions of the text message intervention

***Step 4.1 Final review and revisions in preparation for pilot testing*** Comments from the reviewers were collated onto a spreadsheet of the message delivery plan. Suggestions for revision were collated and incorporated into the intervention. Individual messages were reviewed in the light of comments received and final revisions made, to ensure the complete intervention was coherent. The final intervention included 95 messages to be delivered over 84 consecutive days.

The intervention will be delivered to people with stroke in two waves of pilot testing. Messages will be delivered via TextApp, a software tool developed by one of the research team (CJ), which has been used in several text message behaviour change intervention studies [61, 62, 64]. The TextApp delivery system allows personalisation to be programmed into the message schedule, monitors message delivery and handles replies from participants.

## Discussion

Co-production has led to an empirically and theoretically based intervention to promote and increase physical activity among people with stroke when formal rehabilitation ends. The views and perspectives of people with stroke and health professionals provided important insights into perceived barriers to physical activity and identified strategies to overcome them. The structured, multistep approach [41] highlighted areas for improvement through successive rounds of review, providing an intervention to give people with stroke confidence and motivation to increase physical activity.

The intervention is made up of a discrete set of unique messages that all participants receive. Participants’ names and information about their recent or current physical activity goals are used to personalise some messages. The messages incorporate strategies to maximise meaningful engagement, attention and enactment [41, 65]. The intervention can provide daily contact to promote physical activity and boost motivation at a time when people feel vulnerable.

The intention was to design an intervention that could be easily rolled out within NHS rehabilitation services, if shown to be effective. Text messaging is a low-cost intervention that would appeal to service providers if it could be easily administered without placing a burden on rehabilitation staff. For this reason, a decision was taken to include minimal tailoring to individual participants. Many interventions use tailoring, targeting or personalisation of text message content, but it remains unclear which are effective [28, 31, 55]. For this study, the extensive formative work with people with stroke ensured that themes and topics that were relevant to most people with stroke were identified and incorporated into the messages.

A key feature of this intervention is that it does not prescribe exercises or physical activity that participants should undertake, nor does it provide reminders to perform activities at specific times. It is designed to support behaviour change using proven behaviour change techniques and prompts and progress review [39]. This approach differs from many text message interventions where the aim is to provide reminders or prompts to undertake tasks. In a recently published pilot study, the STROKEWALK study delivered instructional text messages to promote regular walking and functional leg exercises over 3 months [36]. A study by Kamwesiga et al. reported early pilot work, where reminder text messages to perform three daily activities were used as part of a family-centred intervention to support participation in daily activities [28, 29]. In contrast, our co-design processes led to a theoretically guided behaviour change intervention centred on participants’ individual goals, which should better promote and maintain physical activity behaviours.

### Strengths and limitations

The main strength of this study was that collaborative working was used throughout the development of the intervention to ensure that issues that are important to people with stroke were addressed. Incorporating the involvement of key stakeholders at all stages, particularly those with lived experience, maximises the acceptability and potential effectiveness of an intervention [66, 67]. The intervention is theoretically based and uses conventional techniques to increase physical activity and to enhance engagement. Clinical guidelines and rehabilitation professionals were consulted on what behaviours should be addressed to increase physical activity among people with stroke.

The study design has some limitations. CWG methodology [42] could not be used as intended due to restrictions imposed by the COVID-19 pandemic. The planned face-to-face meetings were substituted with online MS Teams meetings. These were well attended and allowed several people, who would be unable to travel due to distance or work commitments, to participate. Changing to online format, especially early in the pandemic when online processes were not well established, meant that the depth of discussion originally intended at CWG meetings was not possible. To mitigate this, the meetings were augmented with one-on-one discussion with some CWG members outside of structured group meetings. To develop discussion of topics raised in online meetings, individuals were contacted by email, or small online meetings were convened.

A second limitation is that the people with stroke who participated in the development process may not be representative of all, despite our attempts to be inclusive. Although some of the people who took part were sedentary, many were physically active, which may have influenced their attitudes and therefore input to a study on physical activity.

The intervention itself may be seen to have limitations. All participants will receive the same text message intervention, apart from a few messages that contain personalised information about the individuals’ experience during rehabilitation. People with stroke form a heterogenous group in terms of demographics, stroke severity and disabilities. Although messages were written to be relevant and accessible to all, some may not be applicable for everyone. However, all participants will be keen to regain their independence, which will increase their ability to relate to the intervention components [49]. Participants will be asked to draw on what is helpful in the messages and disregard what is not relevant. Another potential limitation is that the intervention is partially interactive only. Study participants can respond to messages but will not receive replies. Responses from participants will be delivered to the research team, in real time, by email. Thus, if a participant sends a message that requires action, e.g. a request to stop the messages, the research assistant will be able to take appropriate action. Participants will be briefed about this prior to recruitment to ensure that they are not disappointed when their responses are not acknowledged. Finally, we undertook exploratory research to investigate mobile phone use in people with stroke that indicated that a majority did. Nonetheless, many people with stroke experience cognitive and communication difficulties that may exclude use and participation in this intervention.

## Conclusion

Co-design processes were used to systematically develop a theory and evidence-based intervention. This co-design approach could be used to develop interventions for other heath behaviours and with different populations. The next stages will test the intervention for acceptability, feasibility and effectiveness.

## Supplementary Information

**Additional file 1:** Challenges addressed and solutions obtained through collaborative working

**Additional file 2:** Mapping findings to HAPA constructs and the Maintenance Model and identifying BCTs for intervention delivery

**Additional file 3:** Logic Model

## Data Availability

Data are available on reasonable request. Access to data can be arranged by contacting the study chief investigator, Dr. Jacqui Morris (j.y.morris@dundee.ac.uk), to discuss data sharing, data requirements and conflicts of interest, in line with UK and other regulations, including ethics approvals.

## Abbreviations

BCT: Behaviour change technique
CRT: Core Research Team
CWG: Collaborative Working Group
HAPA: Health Action Process Approach
PPI: Patient and public involvement
SIMD: Scottish Index of Multiple Deprivation
