# Supplementary material for: Keeping Active with Texting after Stroke (KATS): development of a text message intervention to promote physical activity and exercise after stroke
Source: Pilot Feasibility Stud. 2023 Jun 23;9:105. doi: 10.1186/s40814-023-01326-x (PMC10288680; doi:10.1186/s40814-023-01326-x)
Supplement: Supplementary file 1 — Additional file 1: Challenges addressed and solutions obtained through collaborative working [file 40814_2023_1326_MOESM1_ESM.docx]

Additional file 1

Challenges addressed and solutions obtained through collaborative working

| **Challenge/topic to be addressed** | **Knowledge sources** | **Theory/justification** | **Solution** |
| --- | --- | --- | --- |
| Reduce feelings of abandonment and isolation | PPI group | People with stroke say it is good to know they are not forgotten i.e. to know someone is there for them – even though it is just a text message | Send regular friendly, personalised messages from a named person (researcher for intervention development, physiotherapist for a trial or at roll-out) |
| Provide continuity from rehabilitation | Collaborative Working Group (CWG) (therapists, people with stroke)  CWG explored whether therapists would be happy to be named in the text message intervention | Study will have more credibility if it links with the therapists that participants trust | Mention the participants’ rehabilitation therapists on messages to create a link with community rehabilitation |
| Many people lose motivation to continue with prescribed exercise when formal rehabilitation stops | Literature, CWG (people with stroke, therapists)  CWG discussed how information about the types of daily exercises that individuals were asked to continue doing post-rehab could be incorporated into the information. | Encourage participants to continue with exercises and activities recommended by physiotherapists and occupational therapists that are familiar to them | Researcher will liaise with rehabilitation therapist (with participants’ permission) to get details of the prescribed exercises. This information will be inserted into a personalised text message |
| Encourage participants to work towards goals agreed with therapists at the end of rehabilitation | CWG (therapists)  CWG discussed how the participants’ goals going forward from rehab could be included in a text message | Health Action Process Approach (HAPA) | Ask rehabilitation therapists to give details of their participants’ most recent goals for recovery  A message including the therapist’s name and recent goals will be personalised to individual participants |
| Some people with stroke are unsure how to set goals without therapist input | PPI group, CWG  CWG and individual interviews were used to explore goal setting and planning and how to move on to setting personal goals without therapist input | HAPA  Maintenance Model | Encourage people to set goals for activities that would be meaningful and enjoyable.  Explain how to set goals and make plans in text messages  Provide examples of goals and plans in text messages  Reinforce information given in a handbook |
| While there is a desire to return to pre-stroke activities or take up new activities, many people with stroke lack the confidence or are unsure how to proceed | PPI group, people with stroke who were interviewed described how they used goals to take up new activities or return to previous activities (albeit sometimes in a different way) | Maintenance Model  HAPA | Suggest setting goals for activities participants would enjoy (to increase maintenance)  Encourage planning on how to return to pre-stroke activities  Use direct quotes from the study data to model goal setting |
| Social support helps people continue with exercise and physical activity | Interviewees reported that advice and support from people who had similar experiences was credible (feel they are not going through it alone) | HAPA | Regularly mention what other people with stroke have said about common challenges e.g. coping with fatigue |
| Many people with stroke experience loss of confidence and low self-efficacy | Literature, PPI, individual interviews  PPI group discussion and interviews explored how people with stroke can rebuild confidence through perseverance and attaining goals. | HAPA | Encourage participants to reflect on progress regularly and celebrate successes  Give examples from other people with stroke mastering challenging tasks  Encourage people to believe in their own ability and to look for new ways to do things if necessary |
| Encourage outdoor walking | Literature, CWG, interviews | Walking is accessible to most people post-stroke and is one of the best forms of physical activity | Encourage people to walk (safely) outside |
| Walking outside is not possible for everyone | CWG | Concern that people with stroke may disengage if messages were not relevant to them | Offer alternatives to walking e.g. suggest engagement with online resources for physical activity, encourage indoor physical activities |
| Encourage enlisting the support of family members and friends | PPI, literature, CWG | HAPA | Give examples of how family and friends can help (do exercises with you, walk with you, plan activities with you, text or phone to see if you’ve done it) |
| Self-monitoring | PPI, literature | HAPA | Suggest self-monitoring of activities, using methods to suit individuals e.g. diary, phone, activity tracker.  Provide a blank calendar which could be used to set goals and monitor activity |
| Many people with stroke suffer from fatigue which affects ability to be active and deters some | Literature, PPI, interviews  People with stroke advised us that coping with fatigue should be addressed in the messages | HAPA | Include messages about fatigue and how to manage it at different points during the 12-week intervention |
| People with stroke often feel that progress has stopped or plateaued, which can reduce motivation | Literature, PPI | HAPA | Encourage regular reflection on progress. The calendar could be used as a tool. Encourage people to self-monitor activity in a way that is accessible and acceptable. |
| Motivation may subside over time and barriers to being active increase | Literature, PPI, interviews, CWG identified barriers to physical activity post-stroke e.g. bad weather, fatigue, exercising becomes tedious  Explain and model coping planning | HAPA  Maintenance Model | Provide suggestions on how to cope with barriers e.g. indoor activities during poor weather  Model coping planning behaviour with direct quotes from people with stroke |
| Some people forget to be active, some because of cognitive problems, others simply forget | Literature, PPI, interviews | Maintenance Model | Provide prompts to be active without appearing didactic  Encourage habit formation  Provide participants with a diary to record goals and monitor activity  Include a handbook to reinforce topics covered in the text messages |
