# Supplementary material for: Keeping Active with Texting after Stroke (KATS): development of a text message intervention to promote physical activity and exercise after stroke
Source: Pilot Feasibility Stud. 2023 Jun 23;9:105. doi: 10.1186/s40814-023-01326-x (PMC10288680; doi:10.1186/s40814-023-01326-x)
Supplement: Supplementary file 2 — Additional file 2: Mapping findings to HAPA constructs and the Maintenance Model and identifying BCTs for intervention delivery [file 40814_2023_1326_MOESM2_ESM.docx]

Additional file 2

Mapping findings to HAPA constructs and the Maintenance Model and identifying BCTs for intervention delivery

| **Intervention components, HAPA constructs [1], Maintenance model [2]** | **Relevant behaviour change techniques [3]** |
| --- | --- |
| **Introduction and preliminaries to gain engagement**  Gain credibility (clarify that the intervention was designed by University staff, people with stroke, health professionals, experts in stroke rehabilitation)  Use the participants’ first names  Explain what to expect during the intervention period  Engage interest by encouraging interaction i.e. ask questions  Address feelings of abandonment  Emphasise the importance of doing activities that are enjoyable  Provide continuity from rehabilitation (include the name of the participants’ therapist)  Build on rehabilitation and recovery since the stroke (mention goals set with therapists) | 3.1. Social support (unspecified)  9.1. Credible source |
| **HAPA Motivational phase, developing intention**  ***Risk perception***  Discuss benefits of exercise and physical activity after stroke, rather than risks of being sedentary  Emphasise the importance of physical activity to continue recovery and achieve benefits  Encourage physical activity for the prevention of further stroke  ***Outcome expectancies***  Strengthen beliefs about the positive outcomes of being active  Encourage continuing with physiotherapist prescribed exercises and other physical activity  Emphasise that physical activity is something to enjoy, not a chore  ***Action self-efficacy***  Foster intentions to continue with exercises and physical activity to aid recovery  Invite people to reflect on goals already achieved since stroke and the benefits of physiotherapy already experienced since the stroke  Give examples of positive experiences of being active from other people with stroke  ***Social support***  Encourage family involvement  Use quotes from other people with stroke in messages to help people feel they are not in this alone  ***Maintenance***  Introduce the importance of selecting enjoyable activities to ensure long-term maintenance early in the intervention period  Ensure congruence with identity and beliefs (physical activity will help recovery and return to previous lifestyle) | 3.1. Social support (unspecified)  4.3. Re-attribution  5.1. Information about health consequences  5.6. Information about emotional consequences  9.1. Credible source  15.1. Verbal persuasion about capability  15.3. Focus on past success |
| **Volitional phase** (Planning and Action)  ***Goal setting***  Build on goal setting done with/by physios  Move goal setting on to what they want to achieve in life, rather than regaining function  Encourage participants to take responsibility for setting their own goals  Demonstrate how to set goals (explain how to do it, provide examples in messages and the handbook, use quotes from other people with stroke to model the behaviour)  ***Action planning***  Provide instruction on action-planning within messages, the handbook and by using quotes from people with stroke  Direct participants to online classes and exercises  ***Coping planning***  Encourage people to consider possible barriers and how to overcome them  Provide examples of coping planning through quotes from other people with stroke  Encourage participants to develop enjoyable routines that fit with their lifestyle and beliefs  Develop discussion and provide examples in the participant handbook  ***Self-monitoring***  Suggest monitoring physical activity (frequency, time, distance)  Promote the use of the blank calendar provided and suggest alternatives e.g. diary, fitness trackers  ***Action self-efficacy***  Increase beliefs about capability  Encourage reflection on progress already made since stroke  Use quotes from people with stroke to demonstrate improvements in self-efficacy  ***Coping self-efficacy***  Explain and provide examples of coping with high-risk situations (e.g. poor weather, fatigue, exercising becoming tedious, pain or co-morbidities, low mood or depression)  Suggest reflection on capacity to overcome setbacks  ***Social support***  Suggest telling family and friends about the study to gain their approval for increased physical activity and exercise  Encourage participants to consider how family members and friends might support them e.g. offering encouragement, reminders to exercise, walking with them  Use quotes from other people with stroke to offer encouragement and support  ***Habit formation***  Encourage repetition of exercises and walking in same context at same time to establish routines  Suggest being active instead of doing something sedentary at a certain time of day e.g. walk instead of watching a TV programme  ***Maintenance***  Encourage people to increase physical activity gradually  Choose activities that are enjoyable  Encourage self-monitoring and reflection on progress  Demonstrate how to develop strategies to overcome barriers through quotes from people with stroke | 3.1. Social support (unspecified)  6.1. Demonstration of the behaviour  6.3. Information about others’ approval  7.1. Prompts/cues  8.2. Behaviour substitution  8.3. Habit formation  8.7. Graded tasks  15.1. Verbal persuasion about capability  15.2. Mental rehearsal of successful performance  15.3. Focus on past success |
| **Volitional phase** (Maintenance)  ***Self-monitoring***  Encourage continuing with self-monitoring and reflection in the long-term  ***Recovery self-efficacy***  Reassure people that although setbacks will happen, it is possible to get back on track  Encourage people to keep going or get back on track if they are not achieving goals or if they stop being active for a while  ***Coping self-efficacy***  Provide suggestions for ways to cope with barriers such as weather and fatigue  Reinforce the importance of doing activities that are enjoyable  ***Social support***  Encourage continued involvement of family and friends  Provide supportive texts from people with stroke  ***Habit formation***  Emphasise the importance of routines, regular exercise sessions or walking  Continuing with goal setting and self-monitoring when the study is finished  Encourage keeping going when the study is finished  ***Satisfaction with behavioural outcomes***  Encourage reflection on progress and benefits already experienced  ***Physical and psychological resources***  Suggest reflection on the self-determination for progress already achieved  Provide texts from people with stroke to illustrate  Encourage people to be proud of achievements so far | 1.1. Goal setting (behaviour)  1.2. Problem solving  1.3. Goal setting (outcome)  1.4. Action planning  1.5. Review behaviour goal(s)  1.7. Review outcome goal(s)  2.3. Self-monitoring of behaviour  2.4. Self-monitoring of outcomes of behaviour  3.1. Social support (unspecified)  7.1. Prompts/cues  8.7. Graded tasks  8.3. Habit formation  15.1. Verbal persuasion about capability  15.3. Focus on past success |

**References**

1. Schwarzer R, Lippke S, Luszczynska A: Mechanisms of health behavior change in persons with chronic illness or disability: the Health Action Process Approach (HAPA). Rehabil Psychol 2011, 56(3):161.

2. Kwasnicka D, Dombrowski SU, White M, Sniehotta F: Theoretical explanations for maintenance of behaviour change: a systematic review of behaviour theories. Health Psychol Rev 2016, 10(3):277-296.

3. Michie S, Richardson M, Johnston M, Abraham C, Francis J, Hardeman W *et al*: The behavior change technique taxonomy (v1) of 93 hierarchically clustered techniques: building an international consensus for the reporting of behavior change interventions. Ann Behav Med 2013, 46(1):81-95.
