# Supplementary material for: Keeping Active with Texting after Stroke (KATS): development of a text message intervention to promote physical activity and exercise after stroke
Source: Pilot Feasibility Stud. 2023 Jun 23;9:105. doi: 10.1186/s40814-023-01326-x (PMC10288680; doi:10.1186/s40814-023-01326-x)
Supplement: Supplementary file 3 — Additional file 3: Logic Model [file 40814_2023_1326_MOESM3_ESM.docx]

**STROKE SURVIVORS**

-Knowledge of benefits of PA and exercise

-Increased motivation to be active

-Ability to set personal goals meaningful to

their context and plan to achieve goals

-Enhanced self-efficacy for PA, exercise

and physical and functional recovery

activities to meet goals

-Long-term uptake and maintenance of

regular PA and exercise participation

-Continuity of activity with rehabilitation

and recovery goals

**REHABILITATION THERAPISTS**

-Increased knowledge of PA promotion

-Tool to support patients to self-manage

PA and activities for recovery after

rehabilitation

**OUTPUTS**

**MODERATING INFLUENCES**

**IDENTIFIED NEED**

Low levels of PA after stroke.

Recovery potential not met at end of rehabilitation

Feelings of abandonment

Low motivation

**EXTERNAL CONTEXT**

**-**Rehabilitation systems and practices

-Clinical and PA guidelines

-Home environment, physical, social

**MEDIATING PROCESSES**

**OUTCOMES**

Develop and maintain:

-Action Control and Self-regulation

-Satisfaction and enjoyment of new --Behaviour

**Social Support:** Involve others; interactive texts, messages from other survivors

**Self-Monitoring and reflection:** Diary, calendar**,** review goals and outcomes

**Maintenance:** develop self-efficacy for long-term maintenance and habit formation early in intervention and throughout.

**SURVIVOR Level MODERATING INFLUENCES**

-Goal setting experience in rehabilitation

-Physical, cognitive, psychological status, mood,

age, sociodemographic status, fatigue

-Mobile phone use

-Timing post-stroke/rehabilitation

-Past and current experience of PA-

-Access to resources to support PA

**Facilitator. therapist introduction**

**Face to face/telephone session** – goal identification

**Handbook –** explaining intervention

**Diary/Calendar**

**Links to resources** to support recovery and PA

**12 Week SMS intervention**

**Theory of behaviour change** : HAPA

**Behaviour Change Techniques** delivered by SMS

Enhance Coping, Maintenance and Recovery Self-Efficacy

**PROXIMAL**

-Increased participation in PA and

exercise in short and long-term

-Increased frequency, duration and

intensity of PA

-Increased participation in activities

supporting physical and functional

recovery goals

-Improved wellbeing

-Improved quality of life

**DISTAL**

**-**Achievement of personal recovery goals

-Increased fitness

-Improved physical and functional recovery outcomes

-Improved self-efficacy for PA, and exercise and activities for stroke recovery

-Reduced risk of secondary stroke

**MODIFIABLE INTERVENTION CHARACTERISTICS**

-Timing post-stroke/ within rehabilitation

-Flexible person-centred goal-setting process

-Behaviours being targeted and undertaken

-Face to face/remote delivery of introductory session

-Available social support

-Delivery by therapists or facilitators

**INTERVENTION INPUTS**

Develop motivation

Develop Task-self-efficacy

Planning

Find ways to take up and maintain physical activity and post-stroke exercise/activities

Intention

**COMPONENTS of 12 WEEK SMS INTERVENTION**

**Information from credible sources**

**Address Risk Perceptions**: Allay fears, explain benefits of reducing risk of further stroke

**Strengthen Outcome Expectancies:** Strengthen beliefs about benefits of exercise, physical activity and recovery activties

**Enhance Task Self-efficacy:** develop and strengthen beliefs in capability

**Set achievable goals:** set and take responsibility for meaningful PA and recovery goals

**Intervention Components and form of delivery**

**Action Planning:** instruction on planning physical activity routines, link to online classes

**Coping Planning:** Address barriers, develop strategies

Maintenance

**Goal Setting**

**Goal Pursuit**
